# Draft Genome Sequence of the Halophilic Pararhodobacter-Like Strain CCB-MM2, Which Has Polyhydroxyalkanoate-Synthesizing Potential

**DOI:** 10.1128/MRA.01248-19

**Published:** 2019-11-14

**Authors:** Ka-Kei Sam, Nyok-Sean Lau, Go Furusawa, Al-Ashraf Abdullah Amirul

**Affiliations:** a Centre for Chemical Biology, Universiti Sains Malaysia, Penang, Malaysia; b School of Biological Sciences, Universiti Sains Malaysia, Penang, Malaysia; University of Maryland School of Medicine

## Abstract

Pararhodobacter-like strain CCB-MM2 is a halophilic alphaproteobacterium isolated from estuarine sediment collected from Matang Mangrove Forest in Malaysia. Here, we present the draft genome sequence of CCB-MM2 and provide insights into its physiological roles and metabolic potential.

## ANNOUNCEMENT

Pararhodobacter is a genus within the family *Rhodobacteraceae* that is incapable of photosynthesis, separating it from the genus Rhodobacter (1). Thus far, only three isolates from the genus have been classified to the species level, namely, Pararhodobacter aggregans DSM 18938^T^ (1), Pararhodobacter marinus CIC4N-9^T^ (2), and Pararhodobacter oceanensis AM505^T^ (3). *Pararhodobacter*-like strain CCB-MM2 was isolated from Matang Mangrove Forest in Malaysia (4°12′48.06″N, 100°38′49.5204″E) and was part of a study aimed at characterizing the mangrove microbiota (4). Subsequent 16S rRNA gene sequence analysis using the EzTaxon server (5) revealed that CCB-MM2 exhibited 97.98% similarity with *P*. *aggregans* DSM 18938^T^. Phylogenetic analysis based on the 16S rRNA gene sequence constructed using MEGA7 (6) also placed CCB-MM2 in the same cluster as *P*. *aggregans* DSM 18938^T^. Average nucleotide identity (ANI) values between *P*. *aggregans* DSM 18938^T^, *P*. *marinus* CIC4N-9^T^, and *P*. *oceanensis* AM505^T^ and strain CCB-MM2, estimated using the ANI calculator (http://enve-omics.ce.gatech.edu/ani/), were 81.78%, 81.49%, and 77.42%, respectively.

CCB-MM2 was revived from frozen glycerol stock; a single colony from an artificial seawater medium (ASWM) plate was inoculated into ASWM broth and grown with shaking at 30°C (7). Genomic DNA was extracted from the late logarithmic phase culture using the DNeasy blood and tissue kit (Qiagen, USA). A sequencing library was prepared using a Nextera XT DNA sample preparation kit (Illumina, USA), and the constructed library was sequenced on a MiSeq instrument using 250-bp paired-end chemistry. In sum, 3,658,868 raw reads, totaling 835.05 Mb bases (∼100-fold coverage), were generated by the sequencing run. The quality of the raw reads was assessed using FastQC v0.11.5 (https://www.bioinformatics.babraham.ac.uk/projects/fastqc/). Default parameters were used for all software unless otherwise noted. SPAdes v3.6.1 (8) was used for the *de novo* assembly of the sequencing data. The draft genome of CCB-MM2 has a length of 5,149,122 bp with a scaffold *N*_50_ value of 132,033 bp and a G+C content of 65.96% (Table 1). The genome of CCB-MM2 was annotated with Rapid Annotations using Subsystems Technology (9) and NCBI Prokaryotic Genome Annotation Pipeline (10). A total of 4,861 genes were annotated from the draft genome, consisting of 4,804 protein coding and 57 non-protein coding genes (48 tRNAs, 3 16S rRNAs, 2 23S rRNAs, and 4 5S rRNAs).

**TABLE 1:** Genome statistics of *Pararhodobacter*-like strain CCB-MM2

Genome statistic	Value
Genome assembly size (bp)	5,149,122
No. of scaffolds	136
Scaffold *N*_50_ (bp)	132,033
G+C content (%)	65.96
Total no. of genes	4,861
No. of protein coding genes	4,804
No. of RNA genes	57

The gene transfer agent (GTA) system is important for genetic exchange in prokaryotes, and the gene cluster has been described in many other species of *Alphaproteobacteria*, including Rhodobacter capsulatus, the best-studied example (11). Genome annotation revealed the presence of an ∼12-kb head-tail gene cluster in CCB-MM2 that is homologous to the R. capsulatus GTA (RcGTA) gene cluster (12). The partial RcGTA-like cluster of CCB-MM2 consists of 13 open reading frames (*orfg2* to *orfg4* and *orfg6* to *orfg15*) that are homologs of the RcGTA structural genes. Additionally, annotation of the genome indicated that strain CCB-MM2 possesses genes encoding key enzymes involved in polyhydroxyalkanoate (PHA) biosynthesis. Genes encoding β-ketothiolase (*phaA*), NADPH-dependent acetyl coenzyme A (CoA) reductase (*phaB*), and PHA synthase (*phaC*) were found in CCB-MM2, revealing its ability to biosynthesize PHA. The availability of the genome sequence of *Pararhodobacter*-like strain CCB-MM2 improves our understanding of the strain’s metabolic pathways and will inevitably facilitate discovery of its potential in synthesizing industrially useful compounds.

### Data availability.

This whole-genome shotgun (WGS) project can be accessed at GenBank under accession no. LRRR00000000. The raw sequence reads are available at the NCBI Sequence Read Archive under accession no. SRR9593062.
